# Nutraceutical Approaches to Dyslipidaemia: The Main Formulative Issues Preventing Efficacy

**DOI:** 10.3390/nu14224769

**Published:** 2022-11-11

**Authors:** Alessandro Colletti, Andrea Fratter, Marzia Pellizzato, Giancarlo Cravotto

**Affiliations:** 1 Department of Science and Drug Technology, University of Turin, 10124 Turin, Italy; 2 Italian Society of Nutraceutical Formulators (SIFNut), 31033 Treviso, Italy; 3 Department of Pharmaceutical and Pharmacological Sciences, University of Padua, 35122 Padua, Italy

**Keywords:** dyslipidaemia, nutraceuticals, bioavailability, nutritional supplements, bioaccesibility

## Abstract

Currently, the nutraceutical approach to treat dyslipidaemia is increasing in use, and in many cases is used by physicians as the first choice in the treatment of patients with borderline values. Nutraceuticals represent an excellent opportunity to treat the preliminary conditions not yet showing the pathological signs of dyslipidaemia. Their general safety, the patient’s confidence, the convincing proof of efficacy and the reasonable costs prompted the market of new preparations. Despite this premise, many nutraceutical products are poorly formulated and do not meet the minimum requirements to ensure efficacy in normalizing blood lipid profiles, promoting cardiovascular protection, and normalizing disorders of glycemic metabolism. In this context, bioaccessibility and bioavailability of the active compounds is a crucial issue. Little attention is paid to the proper formulations needed to improve the overall bioavailability of the active molecules. According to these data, many products prove to be insufficient to ensure full enteric absorption. The present review analysed the literature in the field of nutraceuticals for the treatment of dyslipidemia, focusing on resveratrol, red yeast rice, berberine, and plant sterols, which are among the nutraceuticals with the greatest formulation problems, highlighting bioavailability and the most suitable formulations.

## 1. Introduction

The nutraceuticals market has grown a lot over the last decade, with a global market of approximately USD 117 billion [1]. Nutraceuticals represent a milestone in the health maintaining strategy of people in the western world: from a medical point of view, these supplements are an interesting and reliable tool to improve bone and cartilage health, restore gut physiology and maintain the overall biochemical efficiency of the cells [2]. At the same time, in the last years, several randomized controlled trials (RCTs) and meta-analysis of RCTs highlighted the potential role of some of these supplements in the prevention of several conditions such as cardiovascular diseases (CVDs) and neurodegenerative diseases as well [3,4,5]. In particular, in the landscape of the large number of nutraceutical products available that essentially cover all the medical fields, not always with proven and ascertained clinical benefits, those dedicated to hyperlipidaemia control and overall reduction of cardiovascular risk (CVR) are the most prescribed from clinicians and the most investigated from a clinical and pharmacological point of view [6]. Despite this huge bibliography regarding clinical effects and the pharmacological mode of action, the association with drugs and meta-analyses appeared in the most authoritative biomedical databases such as Pubmed, Scopus, Embase, Google Scholar, Index Copernicus and others, there is still a question mark and an opaque layer beside the real, unquestionable efficacy of nutraceutical products dedicated to blood lipids reduction and CVR prevention [6,7]. The major part of clinical investigations are indeed poorly conducted, with inadequate cohorts of patients and enrolling criteria, or they simply do not conform to the recognized international clinical trial requirements. Even in the cases of well-designed and well-conducted clinical trials, very often the formulations tested are not fully and correctly analyzed under the aspect of the bioaccessibility and bioavailability of the active compounds delivered. The issue of the poor bioaccessibility and bioavailability of nutraceutical products is still infrequently debated and poorly considered in the perspective of assessing the real efficacy of the formulations tested under clinical trials. Very often the bioavailability profile of notorious molecules used to reduce blood lipids or counteract cardio-metabolic risk such as berberine chloride, resveratrol, monacolins and phytosterols is poorly evaluated and taken for granted [8].

The aim of this review was to analyze the pharmacological and clinical profile of the abovementioned compounds and to highlight the main bioavailability issues related to their oral intake. Although these molecules are widely known and marketed in Europe, they are known to have several formulation issues that may significantly reduce treatment efficacy. The authors investigated, where available, the most reliable and best technological and formulative approaches to ensure the most favourable bioavailability in humans.

## 2. Materials and Methods

A systematic search strategy was conducted for this review in order to identify trials in both the Cochrane Register of Controlled Trials (The Cochrane Collaboration, Oxford, UK) and MEDLINE (National Library of Medicine, Bethesda, Maryland, MD, USA; January 1970 to September 2022). The terms ‘Berberine’, ‘Resveratrol’, ‘Phytosterols’, ‘Plant sterols’, ‘dietary supplement’, ‘Red yeast rice’, ‘Monacolin K’, ‘clinical trial’, and ‘human’ were incorporated into an electronic search strategy. The selected references were further screened for application on dyslipidaemia risk factor. After a general introduction with the description of the main formulative problems regarding the lipid-lowering nutraceuticals, the pharmacodynamic profiles, bioavailability and bionutraceutical strategies to improve bioavailability, clinical proven effects and safety profiles were described for each nutraceutical (red yeast rice, berberine, resveratrol and plant sterols and stanols). The authors of the writing and reviewing panels completed Declaration of Interest forms where real or potential sources of conflicts of interest might be perceived.

## 3. Results

### 3.1. Trans-Resveratrol

**Figure 1:**
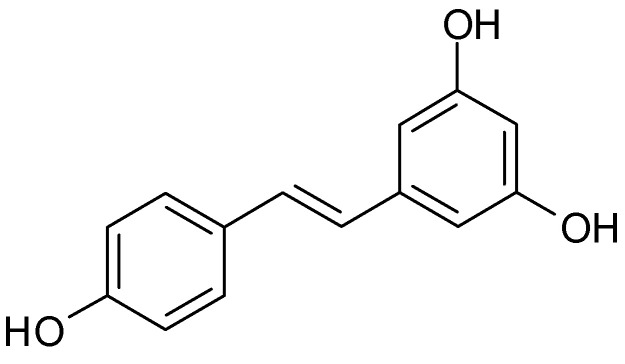
Stilbenic structure of t-Resveratrol.

t-Resveratrol (*t*-Res) (Figure 1) is a stilbenic derived phytoalexin extracted from the peel and seeds of *Vitis Vinifera* and from the roots of *Poligonum Cuspidatum*. It can also be isolated from other vegetal species such as rhubarb (*Rheum rhabarbarum*), apples (*Malus domestica*), blackberries (*Morus nigra*), peanuts (*Arachys hypogea*), pistachio (*Pistacia vera*), cocoa (*Theobroma cacao*), hop plant (*Humulus lupulus*) and jabuticabas or Brazilian grapes (*Myrciaria culifolia*) [9].

This molecule, widely investigated over the last 30 years for its ascertained health boosting properties and famous for playing a pivotal role in explaining the “French Paradox”, represents one of the more effective and potentially promising nutraceutical active compounds. In addition to its well-known properties in CV prevention, due to its anti-inflammatory [10], antidiabetic, [11] antioxidant [12] and lipid-lowering effects [13], there is increasing evidence that t-Res reduces vascular symptoms and bone mass loss during menopause [14,15,16,17]. There is some published evidence that t-Res and its metabolites are capable of inhibiting the growth of colon cancer and preventing its progression in vitro [18], even if data coming from clinical trials in humans is still inconsistent [19,20].

#### 3.1.1. Bioavailability

Despite the numerous and overall well-documented health promoting and pharmacological properties, *t*-Res shows a poor bioavailability after oral administration in humans. The reasons include the massive biotransformation phenomena occurring in the liver microsomes and intestine (CYP3A, CYPB1 and CYPA2), and the relative metabolites are much less active or completely inactive [21,22,23]. In particular, when administered “*per os*”, it suffers a rapid transformation via phase II and some metabolites such as resveratrol 3-*O*-glucuronide, resveratrol 4-*O*-glucoronide, and resveratrol 3-*O*-sulphate can be found in human plasma and urine. For this reason, following oral administration in humans, even the 75% of resveratrol is absorbed (possibly by transepithelial diffusion), the oral bioavailability is low (<1%) through the intestine and liver CYP450 metabolism and probably also by colonic bacteria as well [23,24]. In addition, *t*-Res has poor solubility in water (MW 228.247, pKa 8.99 and LogP 3.4), being about 3 mg/100 mL and it is classified as a class II molecule in the Biopharmaceutical Classification System (BCS).

In a study on 15 healthy volunteers, after a single administration of 500 mg of resveratrol, maximum plasma concentrations (C_max)_ for resveratrol, glucuronated resveratrol and sulphated resveratrol were 71.2 ± 42.4 ng/mL, 4083.9 ± 1704.4 ng/mL and 1516.0 ± 639.0 ng/mL, respectively, while the area under the concentration-time curves from zero to infinity (AUC_0–∞_) values were 179.1 ± 79.1 ng/mL, 39,732.4 ± 16,145.6 ng/mL and 14,441.7 ± 7593.2 ng/mL, respectively [25]. This result was similar to that obtained by Boocock et al. [26].

In addition, *t*-Res pharmacokinetics have shown circadian variation, with higher bioavailability after morning administration [27].

#### 3.1.2. Bionutraceutical Strategies to Improve Bioavailability of *t*-Res

According to this well-defined biochemical evidence, *t*-Res still represents a challenging tool to approach the abovementioned clinical conditions and the contradictory data coming from the published clinical investigations together with the high variability in terms of dosage tested [25], confirming the need to develop new drug delivery systems (DDS) intended to enhance *t*-Res bioavailability [28].

In particular, a reliable delivery strategy consists of promoting *t*-Res absorption through nano-structured delivery systems to improve enteric bioaccessibility [29,30]. The low MW of the molecule, together with the high lipophilicity, makes *t*-Res comply with the fitting rules of sublingual drug absorption, and for this reason, this strategy could potentially change its bioavailability destiny [31]. In this regard, new technological systems, such as liquid spray nanoemulsion, have already shown to be capable of promoting an effective sublingual absorption of lipophilic molecules such as Vitamin D3 [32] and Astaxanthin [33], and could likely represent a further new shining insight dedicated to *t*-Res delivery.

**Figure 2:**
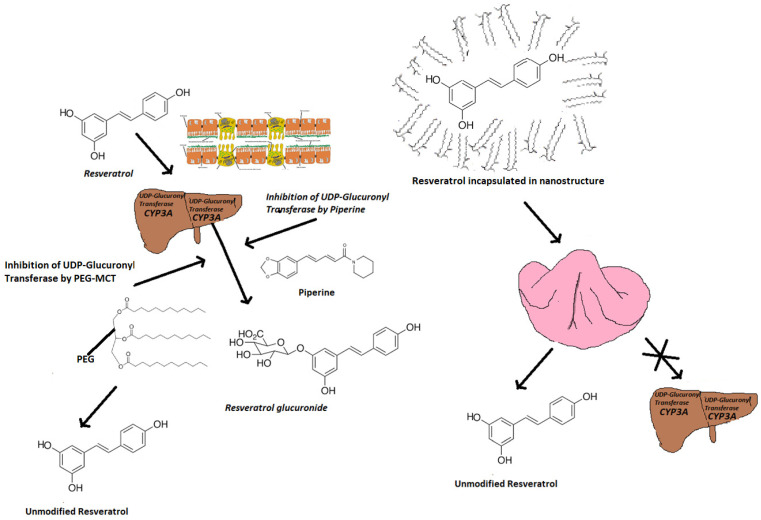
Scheme describing different metabolic pathways of *t*-Res in the presence of UDPGT inhibitors through the enteric (**left**) and sublingual (**right**: with colour pink is represented the tongue) route.

Another promising technological approach described is the association of *t*-Res with UDP-Glucuronyl Transferase (UDPGT) inhibitors (Figure 2). This enzyme expressed both in the enterocyte and hepatocyte is responsible for a massive *t*-Res glucuronidation that strongly limits its bioavailability. Some nano-formulations showed to be able to enhance the overall bioavailability of *t*-RES by inhibiting this enzyme and with specific reference to excipients like PEG-derived middle chain triglycerides (PEG-8-Caprylic/Capric triglycerides, Labrasol, Gattefossè) [34]. These nano-structured delivery systems appear particularly interesting and promising to improve the therapeutic potential of *t*-Res because they enhance both the bioaccessibility and bioavailability of the molecule by respectively promoting their enteric hydro-dispersion and inhibiting UDPGT [35].

The widespread nutraceutical use of alkaloid piperine derived from black pepper, a natural inhibitor of UDPGT, in combination with *t*-Res, seems to be, on the contrary, poorly founded: piperine does not reduce the glucuronides concentration in the plasma of treated patients even though it enhances the overall brain blood flow and cognitive performances with respect to resveratrol alone [36]. This apparent discrepancy is resolved by considering the direct role of piperine in improving blood flow, and as consequence, mild cognitive impairment, as reported [37]. A significant reduction in *t*-Res glucuronidation by piperine has been proven in the animal model (mice) at a dosage of 10 mg/kg of piperine and 100 mg/kg of *t*-Res. Shifting these dosages to a healthy human being of 75 kg/weight, they should be respectively 7.5 g of *t*-Res and 750 mg of piperine. Considering the toxicological falls of repeated assumption of piperine in terms of pharmacokinetics of drugs and its own toxicity, it seems not viable to consider such dosages of this alkaloid. It can even be considered much less as a practicable bioavailability improvement strategy for *t*-Res, especially in the absence of clear proof of evidence in humans.

Interestingly, the assumption of a grape-wine shoot extract containing *t*-Res in human volunteers from a micellar dispersion system has led to a significantly higher bioavailability in comparison with extract alone after oral assumption [38]. A possible explanation of this interesting data is that the micellar dispersion could have boosted the overall enteric bioaccessibility of *t*-Res and the phyto-complex of *Vitis vinifera* composed of polyphenols and flavonoids could have reduced the UDPGT and/or cytochromes enzymes activity [39].

Another possible strategy regards the use of the cubosomes, which are colloids in a stable dispersion of liquid crystalline nanoparticles which improve the bioavailability and stability of drugs that are poorly soluble in water [40]. In particular, the formulation of *t*-Res and piperine-loaded cubosomes have been tested in volunteers with good results [41].

Finally, even the association of *t*-Res with cyclodextrins could represent an important formulative strategy to enhance the solubility of this nutraceutical and thereby improve its bioavailability [42,43].

#### 3.1.3. Pharmacodynamics

*t*-Res is well known for acting through multiple molecular targets. It shows a molecular structure close to that of physiologic and synthetic estrogens and effectively acts as an estrogen-receptor modulating agent [44,45]. It mainly works as a protecting molecule for the cardiovascular system, reducing the aggregation of low-density lipoproteins [46,47]. As reported by Cho et al., *t*-Res could potentiate the lipid-lowering action of pravastatin by down-regulating the 3-hydroxy-3-methyl-glutaryl-CoA (HMG-CoA) reductase [48]. This nutraceutical could also increase the expression of the low density lipoprotein (LDL) receptors in hepatocytes in vitro [49] and decrease LDL oxidation involved in the atherosclerosis process [50]. Another anti-atherogenic action of *t*-Res regards the inhibition of the migration and proliferation of vascular smooth muscle cells [51]

In addition, this molecule appears to activate Sirtuin-1, endothelial nitric oxide synthase (eNOS), nuclear erythroid 2–related factor 2 (Nrf2) and decreases tumor necrosis factor (TNF)-α production, reducing the endothelial apoptosis and vascular inflammation [52]. Finally, *t*-Res has been shown to decrease the expression of adhesion molecules (such as intercellular adhesion molecule-1, ICAM-1, and vascular cell adhesion molecule-1, VCAM-1) via the inhibition of the nuclear factor kappa-light-chain-enhancer of activated B cells (NF-κB) pathway activation [53].

#### 3.1.4. Clinically Proven Effects

The result of RCTs on the lipid-lowering effects of *t*-Res are still contradictory. In a meta-analysis of 21 RCTs, the results indicated that *t*-Res cannot significantly change total cholesterol (TC) (WMD = −0.08 mmol/L, 95% CI: −0.23, 0.08; *p* = 0.349), low density lipoprotein-cholesterol (LDL-C) (WMD: −0.04 mmol/L, 95% CI: −0.21, 0.12; *p* = 0.620), or high density lipoprotein-cholesterol (HDL-C) (WMD: 0.01 mmol/L, 95% CI: 0.04, 0.02; *p* = 0.269) even if the effect on triglycerides (TG) was significant (WMD: 0.58 mmol/L, 95% CI: 0.34, 0.82; *p* < 0.0001) [54]. However, in the recent RCT of Hoseini et al., *t*-Res demonstrated efficacy in patients affected by type-2 diabetes mellitus and coronary heart disease (CHD) in promoting glycaemic control (reduction of fasting glucose (−10.04 mg/dL; 95%CI, −18.23, −1.86; *p*= 0.01), insulin (*p* = 0.01) and insulin resistance (*p* = 0.001) and improvement of insulin sensitivity (*p* = 0.02) compared to placebo), HDL-C levels (3.38 mg/dL; 95%CI, 1.72, 5.05; *p* < 0.001) and total/HDL-C ratio (−0.36; 95% CI, −0.59, −0.13; *p* = 0.002) as well. In addition, *t*-Res also ameliorates oxidative stress parameters such as the total antioxidant capacity (TAC) (*p* = 0.006) and blood concentration of malondialdehyde (MDA) (*p* = 0.04) [55]. However, the dosage of *t*-Res administered was 500 mg daily and patients assumed this dosage for 4 weeks. This dosage is largely higher than the average one generally found in the nutritional supplements available, which ranges from 20 to 100 mg/unit. This data confirms that obtained by the meta-analysis of Hausenblas et al. regarding 196 diabetic patients, even if it emphasized the limitation of the small size of the patient cohorts, duration and variability in dosages [56].

Finally, in addition to red yeast rice (10 mg of monacolins) and a pool of antioxidants (green tea dry extract, coenzyme Q_10_, astaxanthin and quercetin), *t*-Res has been tested in a RCT on 25 moderately hypercholesterolemic patients in primary prevention for CVDs. The results showed an improvement of cholesterol levels (LDL-C, −22.36%; non-HDL-C, −22.83%), high sensible C reactive protein (hsCRP) (−2.33%), and endothelial function (Pulse Volume displacement after monacolin treatment, 18.59%) [57].

#### 3.1.5. Safety Profile

RCTs in humans reveal that *t*-Res is well-tolerated and the adverse events, if any, are mild in severity [24,27]. In general, up to 2.5 g/day, *t*-Res has an excellent safety profile and only high dosages >2.5–5 g/day are associated with mild to moderate gastrointestinal symptoms [58].

### 3.2. Berberine

**Figure 3:**
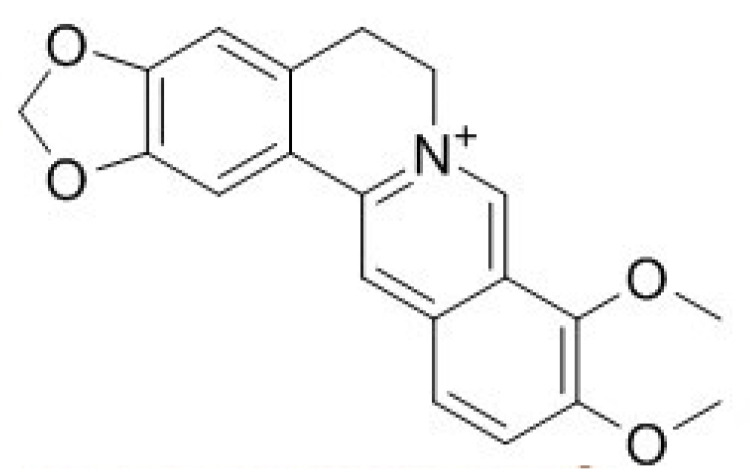
Chemical structure of Berberine.

Berberine (BBR) is a quaternary benzylisoquinolinic alkaloid (Figure 3) present in various plant species such as *Coptis*, *Hydrastis* and *Berberis*. BBR is only one of the alkaloids present in the rhizome, stem, root, fruit and bark of these plants [59] but it is the most studied because it is known to possesses a variety of pharmacological properties in clinical practice [60,61]. In particular, several RCTs have underlined the potential role of BBR in cardiovascular prevention for its anti-hyperglycemic, anti-hyperlipidemic, anti-inflammatory and antioxidant effects [62].

#### 3.2.1. Bioavailability

**Figure 4:**
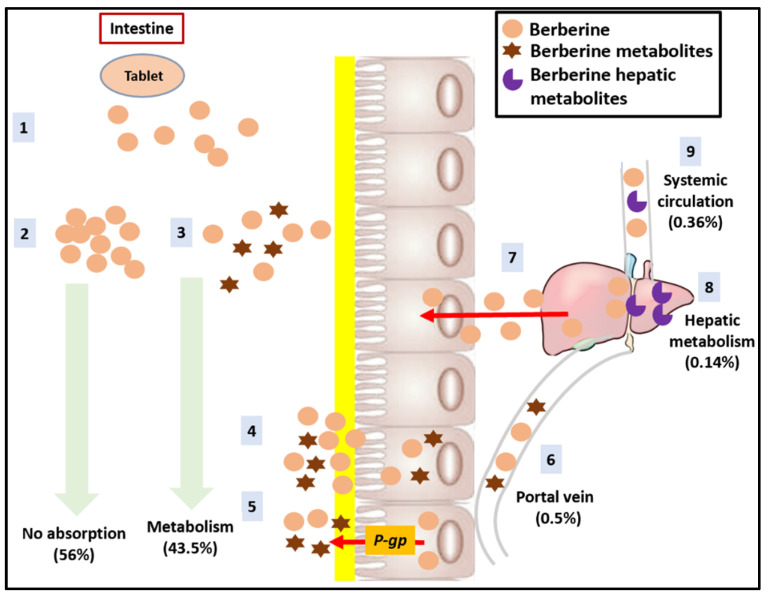
Berberine route after oral supplementation. After ingestion, the tablet disintegrates and releases the BBR particles (1). However, about 56% of BBR is not absorbed in the GI tract due to self-aggregation (2). In addition, intestinal metabolism (operated by both gut microbiota and CYP450) is responsible for the 43.5% of total BBR-particles (3). Finally, the poor permeability (4), P-gp-mediated efflux (5) and hepatobiliary re-excretion (7) also contribute to the reduction of bioavailability. Only 0.5% of BBR enter the portal circulation (6) and 0.36% arrive in the systemic circulation (9).

The most important limitation of BBR in clinical practice regards its absolute bioavailability, which in rats is below 1% (0.36% [63] and 0.68% [64] (BCS class III)). Relatively few studies on humans on the pharmacokinetics of BBR have been conducted: in one of these, regarding 20 volunteers treated with 400 mg of BBR *per os*, the mean C_max_ and AUC_0–∞_ were about 0.4 ng/mL and 9.2 h·ng/mL, respectively [65]. Similar results have been obtained after administration of 500 mg of BBR in 10 volunteers (C_max_ values of BBR and the two metabolites thalifendine and jatrorrhizine: 0.07 ± 0.01, 0.14 ± 0.01, and 0.13 ± 0.02 nM respectively) [66]. The low oral bioavailability of BBR may be due first of all to its poor absorption (56% loss of absorption) and in particular to its self-aggregation, poor permeability, P-glycoprotein (P-gp)-mediated efflux, and hepatobiliary re-excretion (Figure 4) [67]. Self-aggregation is a characteristic of many molecules in the ionized form as BBR; in this form, it easily self-aggregates in the acidic environment of the stomach and upper small intestine with reduction of the solubility (BBR aqueous solubility is about 2 mg/mL), limiting its oral absorption [66]. In addition, BBR has a pH-dependent solubility with an optimal range around pH 7.0 (20-fold higher compared to pH 1.0) [68], and low permeability (effective permeability coefficient (P_eff_): 0.178 × 10^−4^ cm/s) [63]. Lastly, this molecule has been confirmed to be a P-gp substrate expressed in the apical membrane of the epithelial layer of the gut wall: this is an ATP-dependent protein, better known as a multi drug resistance glycoprotein (MDRG), capable of expelling the substrate from the enterocyte towards the enteric lumen (*pumping off*) [69,70].

At the same time, BBR undergoes a marked first pass metabolism in the small intestine (43.5% loss of absorption) and in the liver as well. The mechanisms of intestinal first pass metabolism are still unclear, even if it is supposed that it may mainly be affected by the enzymes CYP2D6, CYP1A2 and CYP3A4 [71,72], and also by the intestinal microbiota [73]. Regarding liver metabolism, BBR is metabolized through phase I oxidative demethylation (with the formation of numerous metabolites such as berberrubine, thalifendine, demethyleneberberine, and jatrorrhizine) followed by phase II glucuronidation (with the formation of BBR glucuronide conjugates). Some of these metabolites remained active on BBR’s targets in the liver but with a reduced potency [74,75].

For these reasons, only about 0.5% of the dose of BBR passes to the portal circulation and arrives into the liver. Of this, another 0.14% is lost with the hepatoenteral circulation process [76].

#### 3.2.2. Biopharmaceutical Strategies to Improve Bioavailability of BBR

In recent years, alternative approaches have been studied to increase the bioavailability of BBR, using (1) permeability enhancers, (2) P-gp inhibitors, and (3) lipid microparticle delivery systems [76].

**Figure 5:**
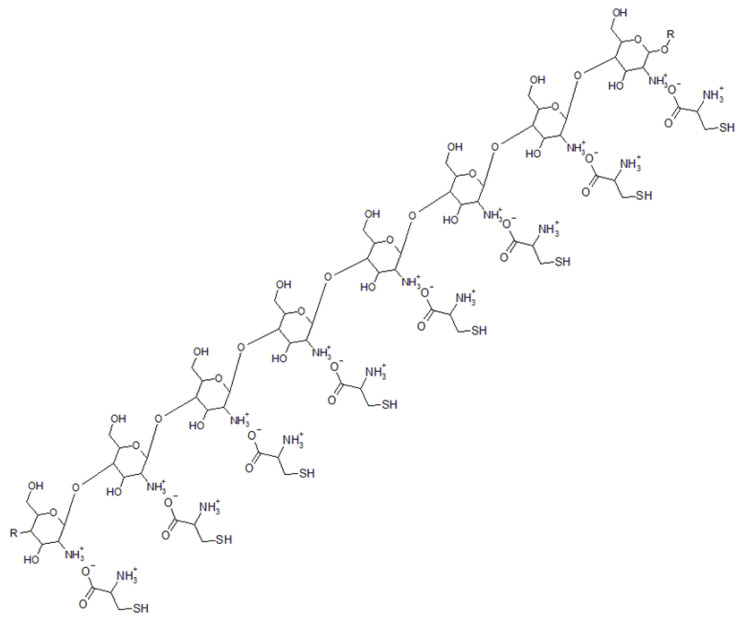
Chitosan cationized by NAC. The carboxylic group of NAC protonates the aminic group of Glucosamine, producing a polycation.

Regarding permeability enhancers, sodium caprate is an anionic surfactant approved by the FDA as an excipient in medical preparations [77]. Its mechanism of action regards first of all the ability to enlarge the intestinal tight junctions [78], in addition to contributing to the formation of BBR–caprate salts, increasing the lipophilicity of BBR [79]. Several preclinical studies have shown that co-administration of sodium caprate increases the bioavailability of BBR, increasing its pharmacological effects [78,80]. Even if sodium caprate can be considered a safe molecule [79], some cases of transient and reversible intestinal damage are reported in the literature [77]. For this reason, in recent years the research has shifted to another interesting molecule: chitosan (CH) (Figure 5). This is a cationic polysaccharide from the shells of shrimps and crabs that displays a dose-dependent enhancement of BBR intestinal permeation (CH is present in formulations at 0.5%, 1.5%, and 3.0%) [81]. CH acts through the inhibition of P-gp (Figure 3), the regulation of tight junctions with the enhancement of paracellular permeability, and its mucoadhesive properties [82]. In particular, the interactions between positive charges expressed by quaternized derivatives of CH (such as trimethyl CH), and the negative charges of the carboxylic groups of tight junction proteins determine the change of the steric tertiary structure of tight junctions, thus increasing permeability [83]. As reported by Fratter et al., CH is a poly-amino sugar soluble in acidic water solution (pH < 5). For this reason, the use of acidic molecules as *N*-Acetylcysteine (NAC) in the formulations determines the formation of a poly-cationic ammonic structure through the interaction between the carboxylic group pf NAC and the aminic group of glucosamine. In addition, NAC also presents a mucolytic activity, reducing the viscosity of the enteric covering mucous layer which represents a limiting factor in the absorption of active ingredients [84].

Biopharmaceutical strategies to implement BBR bioavailability can also involve the use of P-gp inhibitors: in this sense, natural compounds like silymarin from *Silybum marianum* [85] or some excipients as vitamin E derivatives, cyclodextrins and polyethylene glycols have been tested with some success [76]. Finally, one of the most fascinating and promising techniques consists of the use of lipid microparticle drug delivery systems (LMDDSs) such as liposomes, solid lipid nanoparticles, micelles and nanoemulsions [86]. The mechanisms behind these strategies are multiple and include the reduction of particle size and/or the interfacial surface tension, the improvement of solubility and/or permeability in the intestinal tract and/or endocytosis of encapsulated BBR across the intestinal epithelia, and the enhancement of BBR transportation to the lymphatic system or the reduction of self-aggregation. In the last case, the use of a self-microemulsifying drug delivery system that consists in a mixture of oils and surfactants with BBR have been demonstrated to improve BBR bioavailability in pre-clinical models [87].

#### 3.2.3. Pharmacodynamics

One of the most studied actions of BBR is linked to its lipid-lowering activity. The two essential mechanisms by which BBR regulates plasma cholesterol levels seem to be as follows: firstly, it inhibits the proprotein convertase subtilisin/kexin type 9 (PCSK9) through the ubiquitination and degradation of hepatocyte nuclear factor 1 alpha (HNF-1 alpha), which translated means an increase of the hepatic LDL receptor (LDL-R). Secondly, BBR regulates the expression of hepatic LDL-R [88,89] with a post-transcriptional mechanism. In addition, BBR reduces the intestinal absorption of cholesterol, promoting the replacement and formation of new bile acids, and it is also an activator of AMPK, a kinase that is responsible for the increase of fatty acids oxidation and the reduction of the expression of lipogenic genes [90].

#### 3.2.4. Clinically Proven Effects

The lipid-lowering efficacy of berberine at doses between 500 and 1500 mg has been confirmed by several meta-analyses of RCTs: one of the most recent included 27 RCTs and 2569 subjects. BBR intake showed a reduction of the levels of TC (−25.4 mg/dL, *p* = 0.0002), LDL-C (−25.1 mg/dL, *p* < 0.00001) and TG (−34.5 mg/dL, *p* = 0.0001) and an improvement of HDL-C values (+2.7 mg/dL, *p* < 0.00001), with an excellent safety profile [91,92]. Similar efficacy and safety data were also confirmed in another meta-analysis of 14 RCTs that included more than 3000 subjects where BBR was associated with other lipid-lowering nutraceuticals (BBR in combination with red yeast rice, policosanol, astaxanthin, coenzyme Q10 and folic acid) [93]. Compared with red yeast rice or other compounds with a statin-similar mechanism of action, BBR has a greater effect in the reduction of triglyceridemia and glycemia, confirming its specific role in statin-intolerant subjects, patients with metabolic syndrome and/or hypertriglyceridemia [62]. In patients undergoing coronary angioplasty, berberine has been shown to decrease the levels of interleukin 6 (IL-6) (*p* < 0.05) and the monocyte chemoattractant protein-1 (MCP-1) (*p* < 0.05), as well as hs-CRP (*p* < 0.001), ICAM-1(*p* < 0.001), VCAM-1 (*p* < 0.001), and matrix metallopeptidase-9 (MMP-9) (*p* < 0.001) compared to baseline [94]. Finally, BBR has shown a promising prospect in patients with polycystic ovary syndrome (PCOS) with insulin resistance (IR): a meta-analysis of nine RCTs showed no significant difference between BBR and metformin on the alleviation of insulin resistance, on the improvement of glycolipidic metabolism, or on the reproductive endocrine condition [95].

#### 3.2.5. Safety Profile

Based on the studies conducted to date, BBR administration at dosages between 500 mg and 1 g/day could be considered safe and well tolerated. The reported side effects have been mild, mostly of a gastrointestinal nature (diarrhoea, abdominal distension and constipation), and comparable with those in the control group [62]. No significant difference was found in the levels of creatinine, aspartate transaminase (AST) and alanine transaminase (ALT) in comparison to the control group [96], which on the contrary tends to decrease in patients with non-alcoholic liver steatosis [97]. The main contraindications concern pregnancy and breastfeeding (BBR can be transmitted to the new-born through the breastfeeding). In addition, BBR could cross the placenta and might cause harm to the foetus. Finally, kernicterus, or bilirubin encephalopathy, has been found in some infants exposed to BBR: this molecule seems to reduce the hepatic clearance of bilirubin [98,99]. Regarding the risk of drug interactions, this is very low in clinical practice, especially for the low systemic bioavailability of BBR. However, at higher doses than those commonly used as a supplement, it can increase the plasma concentration of cyclosporine [100], and consequently the toxic effects, as well as interacting with the drugs metabolised by CYP450 (3A4) (competitive inhibition). Patients treated with drugs with a narrow therapeutic range and high risk of interaction, such as HIV positive antiretroviral treatment, could be pay attention to co-administration of BBR [101]. No drug interaction has been demonstrated with simvastatin and fenofibrate [102]. The International Lipid Expert Panel (ILEP) recommends the use of BBR in particular in statin-intolerant dyslipidemic patients, precisely because of its high tolerability profile [7,103]. In conclusion, BBR supplementation, in the short-medium term, at dosages between 500 mg and 1000 mg/day, has proven to be safe and well tolerated, even in fragile patients. Further RCTs are needed to confirm the long-term safety profile of this molecule.

### 3.3. Plant Sterols and Stanols

**Figure 6:**
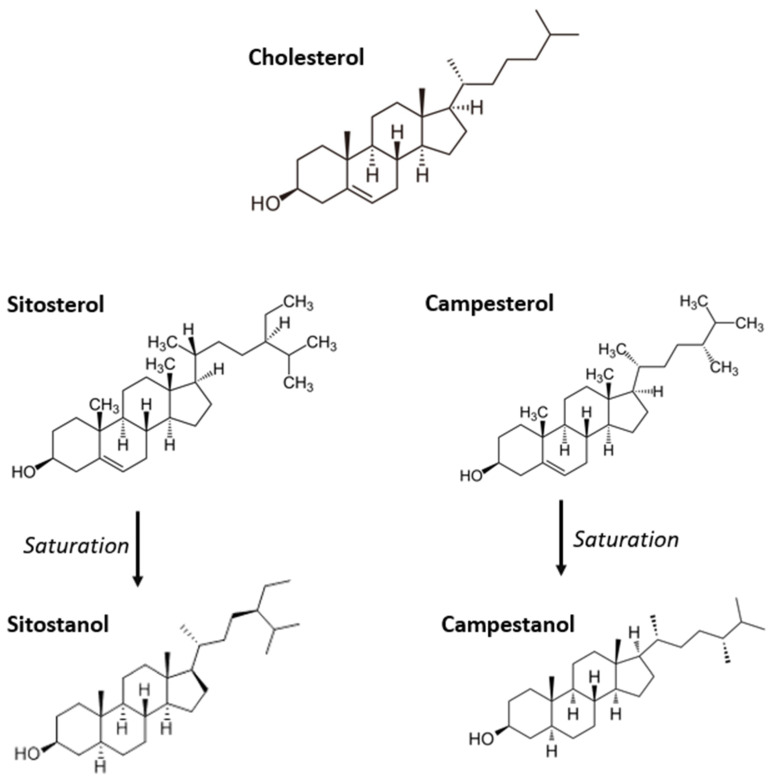
Chemical structure of plant sterols and stanols.

Plant sterols and stanols (PS) are molecules similar to cholesterol that are naturally present as fatty acid esters, hydroxycinnamic acid esters, and glycosides in several plant sources such as cereals, nuts, legumes, seeds, fruits and vegetable oils and fat spreads [104]. In particular, the most abundant PS in western diets are sitosterol (66%), campesterol (22%), stigmasterol (8%), and sitostanol plus campestanol (4%) which are present in the above-mentioned sources [105]. Compared with cholesterol, PS differ structurally in the presence of a methyl (campesterol) or ethyl (B-sitosterol) group in the side chain at C24, or an extra double bond in C22 (stigmasterol), while stanols as beta-sitostanol, stigmastanol and campestanol, are the saturated derivatives of sterols (Figure 6) [106]. Western diets are characterized by a relatively low daily intake of PS (on average 300 mg plant sterols and 17–24 mg plant stanols per day). However, some populations like vegetarians or the Japanese consume up to 600 mg of PS/day [107]. PS are not synthesized endogenously. For this reason, each PS found in the circulation is derived from the diet.

#### 3.3.1. Pharmacodynamics

Once taken with the diet or as supplements, all ester bonds of PS are cleaved by specific enzymes within the gastro-intestinal tract, releasing free PS. The main lipid-lowering mechanism of action of free PS regards the reduction of intestinal cholesterol absorption through the competition in the formation of solubilized micelles [108]. Like cholesterol, PS are taken up from the mixed micelles into enterocytes via the Niemann-pick C1-like 1 (NPC1L1) transporter presents on the brush border membrane [109]. NPC1L1 is well known to be the pharmacological target of ezetimibe, which efficiently reduces the absorption of intestinal cholesterol [110]. After uptake into the enterocytes, while the cholesterol is a substrate for intestinal acyl-CoA cholesterol acyltransferase (ACAT), PS are not easily esterified and thus not efficiently incorporated into chylomicrons. In fact, as a consequence of the reduced esterification, PS are excreted back into the intestinal lumen through the ATP-binding cassette protein family (ABCG5 and ABCG8) shuttles [111]. Recently, another lipid-lowering mechanism of PS proposed regards the “TICE” (trans-intestinal cholesterol excretion) pathway. In particular, PS seem to be able to stimulate the efflux of cholesterol from the brush border membrane into the intestinal lumen [112]. Finally, PS intensify the expression of the ABCA1 transporter and inhibit the ACAT enzyme, reducing the amount of cholesterol absorbed from 50% to 30% [113].

#### 3.3.2. Bioavailability

**Figure 7:**
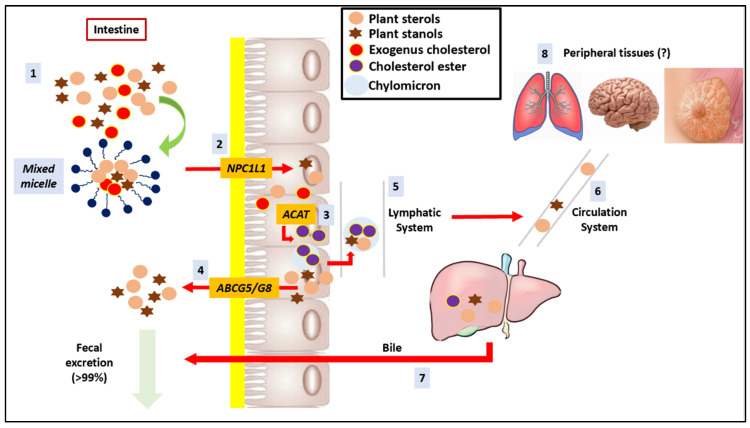
Plant sterol and stanol physiology. (1) PS arrive into intestinal lumen with exogenous cholesterol after a meal. (2) PS are taken up from the mixed micelles together with meal fats and bile and pancreatic secretions, which facilitate the entrance into enterocytes via the NPC1L1 transporter present on the brush border membrane of the enterocyte (3) Into enterocytes, while the cholesterol is a substrate for intestinal ACAT, PS are not easily esterified and thus not efficiently incorporated into chylomicrons (<1%). (4) PS are excreted back in the intestinal lumen through the ATP-binding cassette protein family ABCG5, and ABCG8 shuttles and is eliminated through the faeces with unabsorbed cholesterol. (5) A small percentage of PS is incorporated into chylomicrons and subsequently (6) reaches the bloodstream through the lymphatic system. (7) PS reach the liver, but most of the PS absorbed are re-excreted with the bile and thus eliminated through the faeces. Although circulating concentrations of PS are very low (6), they are probably taken up by several peripheral tissues in the brain, lungs and breasts (8).

Being excreted back into the intestinal lumen through the ABC protein family, the bioavailability of PS is very low (from 0.5% for sitosterol to 1.9% for campesterol compared to 55–60% of exogenus cholesterol). In general, sterols are better absorbed than stanols and campesterol is better absorbed than sitosterol. The reason is that sterols with longer side chains and no double bond have an increased hydrophobicity and thus decreased micellar solubility [114]. Concentrations of PS are 15 to 30 times higher than those of stanols, but 200 times lower than those of cholesterol. After an ingestion of 1.6 g of PS, serum concentrations of sitosterol and campesterol increase on average by 2.2–5.0 μmol/L: however, as highlighted by the meta-analysis of Ras et al., plasma levels of PS showed a dose-dependent trend [111]. The 0.5–2% of PS that enter into circulation are rapidly excreted by the liver and secreted into bile via hepatic ABCG5/G8 (classic paradigm) [115]. In the last years, the researchers have focused on a new paradigm concerning the hypothesis that PS, upon reaching the circulation, are partly distributed to the peripheral tissues (such as lungs, brain and breast) and are also incorporated into red and white blood cells and platelets (Figure 7) [106]. In any case, it is important to emphasize that PS should be administered with a “fat vehicle” such as spreadable fats or emulsification agents like lecithin to improve their dispersion, solubility and incorporation into micelles, and thus to achieve a better lipid-lowering effect [7]. A meta-analysis of RCTs showed that the best fat carriers for PS are rapeseed and canola for their high content of monounsaturated fatty acids, which enhances the functionality of PS [108].

In people with sitosterolemia, a rare recessive disease characterized by mutations of the ABCG5 or ABCG8 transporter genes, the levels of PS can increase excessively from 30 to 100 times higher compared to normal values, especially in heterozygous subjects [114]. Elevated plasma PS levels are also observed in people with the Apolipoprotein E isoform E4 [116]. Conditions such as obesity, insulin resistance and diabetes type 2, metabolic syndrome and familial combined hyperlipidemia (FCH) are associated with a decreased absorption of PS, probably for the increased expression of the ABCB4 (MDR2) transporter gene involved in the biliary excretion of sterols, while the pharmacological treatment with statins induces a compensatory increase in cholesterol absorption but also PS levels as well [114].

#### 3.3.3. Clinically Proven Effects

The lipid-lowering effects of PS have been demonstrated in several RCTs (>120) [117]. In the meta-analysis of Ras et al. regarding 41 RCTs and 2084 subjects, PS at dosages between 300 mg and 3.2 g/day (mean dose: 1.6 g/day), showed a mean reduction of LDL-C of 8.5% (−0.33 mmol/L). PS have been administered through different sources such as yogurt, dressing, bread or mayonnaise for a duration between 21 and 315 days (median duration of the studies: 28 days) [111]. Both the consensus paper of the European Atherosclerosis Society (EAS) and the International Lipid Expert Panel (ILEP) recommends the use of PS, with a daily intake of 2–3 g and an expected average LDL-C reduction of 10–15% [7,106]. The lipid-lowering effect of PS is dose-dependent and proportional up to 3 g/day (above 3 g/day, there is the saturation of the uptake and transport process of cholesterol) with a mean reduction of LDL-C of 12% and no differences in efficacy between sterols and stanols. In patients with hypertriglyceridemia, PS have some impact on TG reduction [118]. Furthermore, PS supplementation seems to be effective in reducing the high-sensitivity C-reactive protein levels [119].

A meta-analysis of eight RCTs showed that the addition of PS to statin therapy induce reductions in LDL-C equivalent to doubling the dose of statins administered (additional reduction of LDL-C of −0.34 mmol/L), suggesting a role of PS in minimizing the side effects of high doses of pharmacological treatment [120].

In an RCT of 21 mildly hypercholesterolemic subjects, the association of PS with Ezetimibe (EZE) 10 mg resulted in significantly lower intestinal cholesterol absorption (598 mg/day, 95% CI 368 to 828) compared to control (2161 mg/day, 1112 to 3209) and ezetimibe alone (1054 mg/day, 546 to 1561, both *p* < 0.0001) [121]. However, the LDL-cholesterol-lowering effect of the PS/EZE seems to not differ from ezetimibe mono-therapy [122], even if Lin et al. reported a possible additive effect PS + EZE compared to ezetimibe alone [121]. These contradictory clinical data introduce the issue of interference between the mechanism of action of EZE and that of PS. EZE works in reducing enteric cholesterol absorption by inhibiting the NPC1L1 protein [123] which is the main carrier of cholesterol internalization placed in the brush border of the enterocyte [112]. This same protein is involved in the internalization of PS into the enterocyte and for this reason EZE theoretically inhibits PS internalization, preventing their ability to compete for Cholesterol absorption. This fact has been clinically proven in patients with sitosterolemia [124,125]. More and wider clinical investigations should be carried out to ascertain if and how this pharmacologic issue can interfere with the additive hypo-cholesterolemic effect of the association of EZE + PS. Nerveless pharmacologic investigations should be addressed to understand in depth the mechanistic phenomena governing this interference and whether up-regulation mechanisms take place.

#### 3.3.4. Safety Profile

The use of PS as lipid-lowering nutraceutical has been approved by various regulatory agencies such as the European Food and Safety Authority (EFSA), the U.S. Food and Drug Administration (USFDA), and Food Standards Australia New Zealand (FSANZ). PS have shown a good safety profile in the middle term even if data for treatment longer than two years are still not available [126]. The meta-analysis of Baumgartner et al. (41 RCTs and 3306 subjects) showed that the intake of PS (a mean of 2.5 g/day) reduced the total cholesterol-standardized concentrations of β-carotene (−10.1%, 95% CI: −12.3; −8.0), α-carotene (−7.8%, 95% CI: −11.3; −4.3), and lycopene (−6.3%, 95% CI: −8.6; −4.0) [127]. Plasma retinol (vitamin A) and vitamins D and K are not significantly affected [104,128]. Finally, PS administration is contraindicated for individuals diagnosed with sitosterolemia; these patients usually exhibit tendon xanthomas and premature atherosclerosis [129].

### 3.4. Monacolins from Red Yeast Rice (RYR)

RYR is the product of the fermentation of the fungus *Monascus* spp. on rice (*Oryza sativa* L.) and it is widespread, with some variations throughout Asia where it has been used for over a thousand years as a food and as a medicinal remedy [130,131,132] and more recently as a food coloring agent [133,134]. It is also known for and used as a medicine belonging to TCM (Traditional Chinese Medicine) to promote blood circulation and problems related to gastric disorders [134,135].

**Figure 8:**
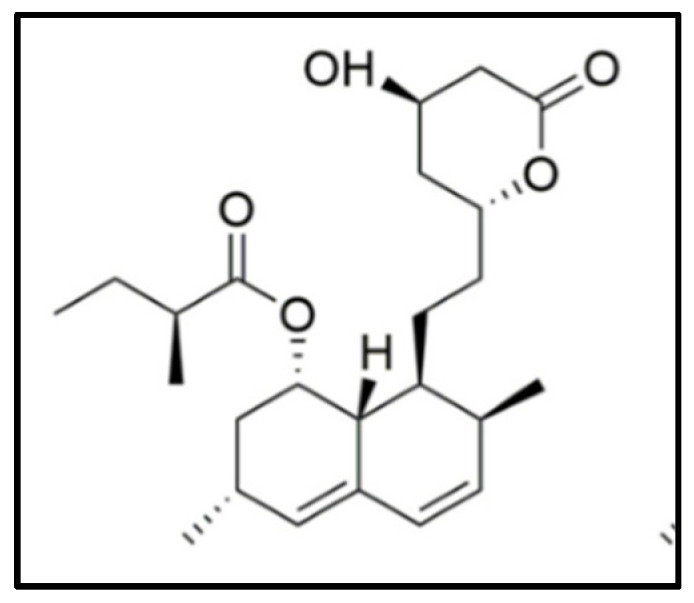
Molecular structure of Monacolin K, also known as Mevinolin or Lovastatin.

RYR has been and still is the subject of numerous studies aimed at investigating and explaining its activities starting from its millenary uses in traditional medicine. In particular, the attention towards this compound started after the isolation among its components of mevinoline, better known today as monacolin K (Figure 8), or Lovastatin, previously isolated from a matrix fermented by *Aspergillus terreus* [136].

From a phytochemical point of view, RYR includes several classes of compounds including monacolins, pigments, organic amino acids, sterols, decalin derivatives, flavonoids, lignans, coumarins, and terpenes [135]. Referring to the monacolins family, a total of 23 monacolins have been isolated and identified in RYR, including Monacolin K, J, L, M, X [130], and Q, R, S, in addition to their methyl esters and hydroxyacid forms (Table 1) [130,137]. On the other hand, 25 different pigments have been isolated, and many of them, beyond their coloring capacity, have shown health-related activities including the lipid-lowering and anti-inflammatory ones [138,139].

The sterolic portion of the phytocomplex, consisting of nine sterols including beta-sitosterol and stigmasterol, also showed a lipid-lowering activity [140,141]. It is possible that non-statin components of RYR exert pleiotropic actions as lipid-lowering agents, thus reaching the same values as the groups treated with single component statins [142,143].

#### 3.4.1. Bioavailability

Monacolin K is a lipophilic drug that presents problems of solubility and permeability (class II BCS). The lipophilic nature of the molecule (solubility equal to 1.3 µg/mL in water [135,144]) makes it poorly bioavailable after oral administration without a correspondence between the absorbed dose and the available one. In fact, it is absorbed for 60–80%, but only 5% is really available due to extensive metabolism in the gut and in the liver, and transmembrane efflux via P-gp pump [135,144,145]. The main cytochrome responsible for the metabolism of monacolin is the CYP450 3A [146]. However, it is noted that the pharmacokinetics of lovastatin are different when administered individually compared to administration as a component of RYR [147]. Indeed, some studies, performed on Caco-2 models, have highlighted that RYR extract had a greater dissolution rate and therefore bioavailability, compared to monacolin K alone. Chen et al. have speculated that this was due to an ability of the RYR extract to inhibit the activity of CYP450 and the P-pg pump [148,149]. In particular, in two human studies, Chen et al. showed that in the volunteers who received RYR, AUC and a Cmax was higher for both Monacolin K and its active acid form, and Tmax was lower compared to the group that received Lovastatin alone, thus concluding that the oral bioavailability of Lovastatin is enhanced when administrated as part of the RYR due to a higher dissolution rate. These data, which show a higher bioavailability of monacolin K from RYR compared to the monacolin drug, have been confirmed by several clinical studies which show that the intake of 5–6 mg/day of Monacolin K from RYR are equivalent for efficacy to 20–40 mg/day of monacolin K alone [149,150,151].

The main hypothetical causes for these differences in pharmacokinetics between the two forms of Monacolin are:

- The presence of other monacolins within the phytocomplex that may work synergistically with lovastatin
- The higher dissolution rate of monacolin K within the phytocomplex compared to the pure molecule
- An inhibitory interaction by some components of the phytocomplex with cytochromes and P-gp pumps [149,150,151].

#### 3.4.2. Biopharmaceutical Strategies to Improve the Bioavailability of Monacolin K

Based on the data described above, the bioavailability of monacolin K can be improved with different strategies. One of these may be the formulation of monacolin K in association with components that inhibit the action of the P-gp pump and cytochrome [146]. In some studies, an improved bioavailability of monacolin K has been demonstrated in conjunction with grapefruit juice, whose flavonoids are known to have an inhibitory activity against CYP450 3A. In particular, in an open, randomized, two-phase crossover study, Kantola et al. found that the Cmax and the AUC of monacolin K with grapefruit juice was increased 12-fold (range, 5.2-fold to 19.7-fold; *p* < 0.001), and 15-fold (range, 5.7-fold to 26.3-fold; *p* < 0.001), respectively [152].

A second strategy aimed at improving the bioavailability of monacolin is to increase its dissolution rate [152,153]. For this purpose, lipid formulations consisting of an active principle solubilized in triglycerides, partial glycerides, surfactants or co-surfactants can be used [154]. Chen et al. have highlighted that the release of lovastatin from tablets was highly dependent on the dissolution condition (in particular on the dissolution medium) and, in the presence of Sodium Lauryl Sulphate (SLS) it was 180-fold higher than in an acetate buffer. It is likely that lovastatin’s release was also increased using simulated intestinal fluid containing taurocholate and lecithin [149].

These kind of formulation have the advantage of being versatile, as they can come in the form of solutions, suspensions, emulsions, self-emulsifying systems and microemulsions and of being able to exploit the lipid metabolism and the lymphatic pathway for their absorption into the intestine [155]. The intestinal lymphatic pathway has a fundamental role in the absorption of lipid substances (e.g., long chain fatty acids, fat soluble vitamins) and therefore can also be important for lipophilic drugs. In fact, while hydrophilic active ingredients, administered orally, have access to the systemic circulation via the portal pathway, the highly lipophilic ones can reach the circulation directly using the lymphatic pathway [155]. The main advantage of drug absorption through the intestinal lymphatic system is the bypassing of the hepatic first pass mechanism which, as regards monacolin, is the main obstacle to its absorption. The disadvantage, on the other hand, can be identified in the fact that these formulations, being in liquid form, can present stability problems [154]. A solution to these problems is represented by the possibility of supporting these formulations on solid carriers in order to produce solid pharmaceutical forms for oral use. Regarding solid forms, the strategy of using hydrophilic carriers has also given good results in terms of the improved bioavailability of lovastatin. Wu et al. have shown that with in vitro and in vivo studies that loading of monacolin on a biodegradable porous starch foam with a nanoparticle structure can effectively improve the bioavailability of the molecule [153].

#### 3.4.3. Pharmacodynamics

**Figure 9:**
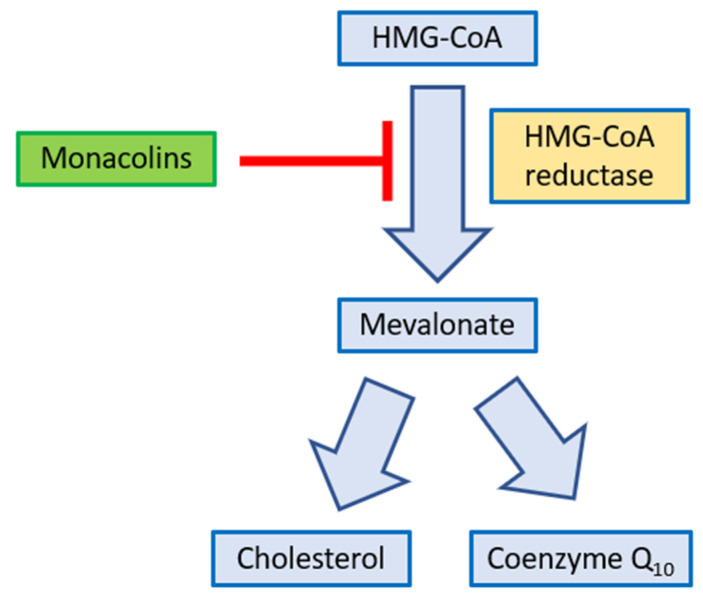
Hypolipidemic mechanism of action of monacolins.

From the physical-chemical point of view, in their active form all HMGRIs (HMG-CoA Reductase Inhibitors) have a carboxyl group, which is a necessary functional group for inhibitory activity. It has a pKa between 2.5 and 3.5 and at physiological pH it is mainly ionized. It’s considered as a neutral lactonic prodrug. Once ingested it is hydrolized to its acid form, which is responsible for the pharmacological activity of the drug [135,156]. The results of subsequent metabolic reactions show no pharmacological activity. The mechanism of action behind RYR cholesterol-lowering action is due, as is now known, to the presence of monacolins, so-called real “natural statins”. These compounds, which naturally constitute about 0.4% of red yeast rice, and up to 1.9% in nutraceutical extracts, competitively inhibit HMG-CoA reductase, reducing cholesterol biosynthesis [150]. As shown in Figure 9, HMG-CoA reductase is the enzyme which mediates the reaction from HMG-CoA to mevalonate. The inhibition of the activity of this enzyme results in a decrease of the production of endogenous cholesterol.

The RYR phytocomplex also contains sterols, including beta-sitosterol, campesterol, stigmasterol, and sapogenin, isoflavones and isoflavone glycosides, and mono- and poly-unsaturated fatty acids [130,150]. These phytosterol compounds, isoflavones, and fatty acids may inhibit cholesterol absorption or increase the clearance of cholesterol from the circulation [157]. Although it has been proven that the main responsible molecules for the cholesterol lowering action of RYR are the monacolins, it has been observed that even the fraction of yellow, orange and red pigments is able to positively and significantly influence the metabolism of lipids. This improves blood levels and also suppresses the hepatic accumulation of lipids with a consequent decrease in steatosis, with the promotion of the faecal elimination of cholesterol, triacylglycerol, and bile acids. The mechanism of action for these molecules consists of an up-regulation of farnesoid X receptor and peroxisome-proliferator-activated receptor-gamma levels, the main receptors for the metabolism of cholesterol and the homeostasis of bile acids [135,138].

#### 3.4.4. Clinically Proven Effects

In the last thirty years, many clinical studies have been performed with the aim of demonstrating the effectiveness of RYR and in particular of its monacolin constituents, in the control and contrast of hyperlipidaemias and more generally of dyslipidaemias, and still today scientific research is focused on the determination of the correct dosage of the substance and its long-term safety [135].

The hypolipidemic potential of RYR has been consistently investigated and proved in several meta-analysis of RCTs: in one of the most recent, including 20 RCTs, the supplementation with RYR (1200 mg–4800 mg/day containing from 4.8 mg to 24 mg of monacolin K), for 2–24 months, has been associated with a reduction of LDL-C (−1.02 mmol/L, range −1.20 to −0.83) compared to placebo, which was not different from moderate-intensity statins (40 mg of pravastatin, 10 mg of simvastatin, 20 mg of lovastatin; 0.003 mmol/L; range, −0.36 to 0.41). It is possible that non-statin components of RYR (such as polyunsaturated fatty acids and beta-sitosterol) exert pleiotropic actions in reducing cholesterol, thus reaching the same values as the patients treated with moderate-intensity statins [158]. In addition, the results showed an increase of HDL-C (0.007 mmol/L; range, 0.03–0.11) and a decrease of TG (−0.26 mmol/L; range, −0.35 to −0.17) compared to placebo. Concerning the safety profile, no significant differences have been observed between active and placebo groups [158]. These data confirmed the results obtained in the Chinese meta-analysis by Liu et al. that included 93 RCTs and a total of 9625 participants [159].

In another RCT that included 4.870 Chinese subjects, the administration of 2.5–3.2 mg of Monacolin K, after a four-week initial period with controlled diet and suspension of lipid-lowering agents assumption, in patients which had a myocardial infarction (MI) within 60 months before enrollment, showed a significant decrease in frequency of major coronary events such as nonfatal MI and death from coronary or cardiac causes when compared to the placebo group (–10.4% and –5.7%, respectively; *p* < 0.001). In addition, another effect highlighted in this study was the decrease in the need for coronary revascularization compared to placebo recipients, which in this case was 33% lower (*p* = 0.004) [160]. Similar results were obtained in a large trial including 1445 patients in secondary prevention with a history of MI (aged 65–75 years). In particular, the supplementation with RYR was associated with a reduction in the risk of coronary heart disease (CHD) (−31.0%; *p* = 0.04), all-cause mortality (−31.9%; *p* = 0.01), stroke (−44.1%; *p* = 0.04), the need for a coronary artery bypass graft, or a percutaneous coronary intervention (PCI) (−48.6%; *p* = 0.07), and malignancies (−51.4%; *p* = 0.03) [161].

Finally, RYR also improves endothelial function and arterial stiffness in patients with dyslipidaemias, measured as flow-mediated dilatation (FMD) and pulse-wave velocity (PWV) [162], in addition to reduction of the serum levels of apolipoprotein B, matrix metalloproteinases 2 and 9, and high-sensitivity C-reactive protein [147].

#### 3.4.5. Safety Profile

The safety profile of RYR is similar to that of low-dose statins [134]. RYR might cause some adverse effects, since monacolin K administration is linked to an increased risk of myopathy [163], symptomatic hepatitis [164], peripheral neuropathy [165], and erectile dysfunction [166]. Among these, the most frequent are myopathy and hepatitis [135]. Mazzanti et al., analysed the adverse reactions of food supplements containing RYR using Italy’s WHI-UMC system and CIOMS/RUCAM score. In particular, they found that 52 out 1261 studies reported adverse reactions to RYR supplementation from April 2002 to September 2015, and myopathy and hepatitis represented the 52.73% of the total adverse reactions [134]. However, although the chronic administration of monacolins could be responsible for mild to moderately severe side effects, it is usually well tolerated, at least at dosages up to 10 mg/day of monacolin K, as highlighted by a recent meta-analysis of 53 RCTs and a total of 8535 patients [167]. RYR seems thus to be an overall tolerable and safe lipid-lowering dietary supplement, especially in patients previously intolerant to statin treatment and at a dosage of 3 mg/day [103].

Due to the statins metabolism by CYP450 3A4, the coadministration of inhibitors or inducers of CYP450 may cause alterations of plasma concentrations of drugs (niacin, cyclosporine, antifungals, fibrates, macrolides, coumarin, verapamil, nefazodone, human immunodeficiency virus protease inhibitors) and nutraceuticals (e.g., grapefruit juice). This increased exposure to the drug may increase the risk of myotoxic side effects and in some rare cases can potentially lead to a statin-induced rhabdomyolysis [135,135].

Companies providing RYR products on the market should be aware of these adverse reactions in order to give consumers proper indication of use and warnings before the assumption of RYR-based food supplements.

Related to RYR safety, particular attention must be paid to the presence of citrinin. Citrinin is a polyketide, secondary metabolite found in several fungi, including *M. purpureus* [130]. This molecule has been found, when ingested chronically, to exert a nephrotoxic activity in different animal models, gradually leading to hyperplasia of the renal tubular epithelium, renal adenomas, and in some cases to renal tumors (at a dose of 50 mg/kg body weight [b.w.] causing tumors in 100% of the animals tested) as well as a disruptive action on metabolic processes in the liver [168].

Citrinin may be contained in the 80% of products with RYR [169]. For this reason, Citrin, as a component, has been involved in several controversies related to the safety of products containing RYR [135]. The EFSA has established 0.2 mg/kg b.w. per day as the highest quantity of citrinin that can be taken by humans with no nephrotoxic effects. However, at these doses, genotoxic and carcinogenic effects are not excluded. In the market, RYR supplements with levels of citrinin exceeding 114 mg/capsule were detected, and at four capsules/day (the recommended dosage) the mean was 456 mg/day of citrinin, which is well above the level of 20 mg/kg b.w. per day suggested by the EFSA [170].

In summary, based on the available clinical data about the lipid-lowering effect of RYR, this nutraceutical can be recommended in patients with moderately high hematic levels of cholesterol.

For evidence of this, the EFSA expressed a positive scientific opinion on the substantiation of health claims about the relationship between assuming RYR and the maintenance of normal plasma LDL-C levels, linked to a dose of 10 mg/die (maximum allowed in Europe) of Monacolin K. However, due to safety reasons, recently some National Regulatory Agencies in Europe have suggested that a lower dosage of Monacolin K is recommended.

## 4. Discussion

CVDs are the leading cause of mortality and disability worldwide, being responsible for up to 31% of deaths (taking an estimated 17.9 million lives each year) in 2012 [171]. In particular, ischemic heart disease and atherosclerosis are the main causes of premature death in Europe and are responsible for 42% of deaths in women and 38% of deaths in men under 75 years old [172]. The global economic impact of CVD is estimated to have been US $906 billion in 2015 and is expected to rise some 22% by 2030 [173]. Among the CVR factors, elevated TC (>5 mmol/L) and LDL-C (<3 mmol/L for patients at low and moderate risk for CHD, <2.6 mmol/L for patients at high risk and <1.8 mmol/L for patients at very high risk [174]) are the major modifiable risk factors for CHD, whereas high concentrations of HDL-C in certain conditions are considered protective [175]. However, LDL-C is considered a fundamental CVR and one of the main targets of both nutraceutical and drug therapies [176]. Many RCTs and meta-analysis of RCTs have shown a relationship between a decrease in the levels of LDL-C and a reduction in the relative risk of CVD [177]. In particular, in a report from the Cholesterol Treatment Trialists’ (CTT) Collaboration regarding >170,000 participants, it was stated that, with lipid-lowering therapy, each further reduction of LDL-C by 1 mmol/L (≈40 mg/dL) decreased the risk of revascularization, coronary artery disease, and ischemic stroke [178] by approximately one-fifth. A reduction of 1 mmol/L is achievable through lifestyle improvements associated with lipid-lowering nutraceuticals. In fact, based on current knowledge, nutraceuticals (mostly BBR, RYR and PS) could exert significant lipid-lowering activity especially in primary prevention, and/or in patients with statin-associated muscle symptoms, or persons already treated with statins and/or ezetimibe but who have not reached the targeted LDL-C level although they are not too far from it [7]. Even the European guidelines for dyslipidemia management consider the possibility of using some lipid-lowering nutraceuticals in clinical practice [174]. In fact, the lipid-lowering nutraceuticals can act through different mechanisms of action, contributing to the reduction of lipid-induced vascular damage, making them potential candidates for improving the lipid-lowering effects when used in combination with diet, drugs, or other nutraceuticals [179]. Nevertheless, the whole nutraceutical sector and in particular nutraceuticals with lipid-lowering action has still strong limits. In fact, while pharmaceutical products are strictly regulated (pre-clinical and clinical research with both in vitro and in vivo studies) and have a governmental sanction [180], nutraceuticals are considered as “foods” and consequently do not require clinical trials, but exclusively a “significant consumption history” on the territory.

For this reason, several products on the market do not possess the minimum requirements with regard to quality, safety and. The most common errors include the combinations of underdosed substances or active ingredients with poor bioaccessibility and bioavailability on which bionutraceutical research has not been carried out. In fact, the success of a pharmacological or nutraceutical treatment does not depend exclusively on the correct choice of the active ingredient and the dosage of administration, but also on the correct bionutraceutical formulation. In the future, in relation to the extensive use of these products, nutraceuticals should be regulated in a defined class. In the USA, due to the lack of a definition, nutraceuticals are regulated as “drugs, food ingredients and dietary supplements” and according to FDA, the label of any products should state that “*This statement has not been evaluated by the FDA. This product is not intended to prevent, cure or treat any disease*” [181]. In Europe, the European Food Safety Authority (EFSA) acknowledged the nutraceutical terminology and is responsible for evaluating the health claims of the substances [182].

## 5. Conclusions

In summary, lipid-lowering dietary supplements have been shown to significantly improve the lipid profiles both alone and in combination with standard pharmacological treatments. However, it must be clearly emphasised that there are still no studies demonstrating that nutraceuticals can prevent CVD morbidity or mortality. In addition, new studies on the bioaccesibility and bioavailability of these supplements, as well as new longer and larger RCTs, are urgently needed both to evaluate efficacy on outcomes and to improve pharmacokinetic parameters.

## Figures and Tables

**Table 1:** Chemical Structures of main monacolins from Red Yeast Rice.

Structures	Molecules	R
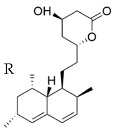	Monacolin K (MK)	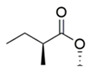
	Monacolin J (MJ)	-OH
	Monacolin L (ML)	-H
	Monacolin X (MX)	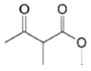
	Monacolin M (MM)	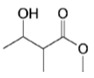
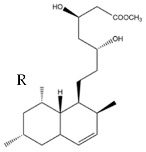	MK Hydroxy acid	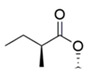
	MJ Hydroxy acid	-OH
	ML Hydroxy acid	-H
	MX Hydroxy acid	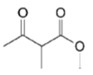
	MM Hydroxy acid	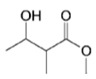
